# Unlocking the potential for optic nerve regeneration over long distances: a multi-therapeutic intervention

**DOI:** 10.3389/fneur.2024.1526973

**Published:** 2025-01-09

**Authors:** Zhen-Gang Liu, Lai-Yang Zhou, Yong-Quan Sun, Yi-Hang Ma, Chang-Mei Liu, Bo-Yin Zhang

**Affiliations:** ^1^Department of Orthopaedics, China-Japan Union Hospital of Jilin University, Changchun, China; ^2^Key Laboratory of Organ Regeneration and Reconstruction, Institute of Zoology, Chinese Academy of Sciences, Beijing, China; ^3^Savaid Medical School, University of Chinese Academy of Sciences, Beijing, China; ^4^Institute for Stem Cell and Regeneration, Chinese Academy of Sciences, Beijing, China; ^5^Beijing Institute for Stem Cell and Regenerative Medicine, Beijing, China

**Keywords:** optic nerve, axon regeneration, retinal ganglion cells, immune microenvironment, multiple genes

## Abstract

Retinal ganglion cells (RGCs) generally fail to regenerate axons, resulting in irreversible vision loss after optic nerve injury. While many studies have shown that modulating specific genes can enhance RGCs survival and promote optic nerve regeneration, inducing long-distance axon regeneration *in vivo* through single-gene manipulation remains challenging. Nevertheless, combined multi-gene therapies have proven effective in significantly enhancing axonal regeneration. At present, research on promoting optic nerve regeneration remains slow, with most studies unable to achieve axonal growth beyond the optic chiasm or reestablish connections with the brain. Future research priorities include directing axonal growth along correct pathways, facilitating synapse formation and myelination, and modifying the inhibitory microenvironment. These strategies are crucial not only for optic nerve regeneration but also for broader applications in central nervous system repair. In this review, we discuss multifactors therapeutic strategies for optic nerve regeneration, offering insights into advancing nerve regeneration research.

## 1 Introduction

The optic nerve transmits signals from RGCs to the brain’s visual processing regions, a pathway that does not regenerate when injured or in degenerative diseases such as glaucoma. RGCs play a crucial role in receiving the initial light signals and transmitting them to the brain’s visual perception process. The layered structure of the retina consists of different cellular arrangements. The nuclei of photoreceptor cells, particularly the rods and cones, are located in the outer nuclear layer (ONL), while the nuclei of various interneurons including amacrine cells, bipolar cells, and horizontal cells are mainly located in the inner nuclear layer (INL). RGCs are situated in the ganglion cell layer (GCL), and their axons project through the nerve fiber layer (NFL). RGCs constitute approximately 1% of the total number of cells in the human retina (about 1.2 million) (1). RGCs consist of a cell body, dendritic structure, and a single axon. The dendrites are essential structures for receiving signal inputs from bipolar and amacrine cells, converting the signals from photoreceptors into action potentials, and conducting them along the long axons, ultimately reaching the lateral geniculate nucleus (LGN) in the thalamus, which then relays the signals to the cerebral cortex. The retina contains multiple types of RGCs, which can be distinguished based on their unique morphological and physiological characteristics. Single-cell RNA sequencing has revealed over 46 distinct subtypes of RGCs in the adult retina (2), yet they are fundamentally similar in overall morphology and synaptic structure. Each specific subtype of RGC contributes differently to various aspects of visual perception. Axonal regeneration is only observed in less than 10% of RGCs after retinal injury, and these neurons are primarily *α*-RGCs. They have larger cell bodies and larger dendritic branches, and they have fast axonal conduction capabilities, transmitting visual information to the brain more quickly than other RGCs (3).

Optic neuropathies primarily include inflammatory optic neuropathy, ischemic optic neuropathy, toxic/nutritional optic neuropathy, hereditary optic neuropathy and traumatic optic neuropathy (4). Traumatic optic neuropathy like Optic nerve crush (ONC) leads to a rapid and transient influx of Ca2+ from the extracellular space into the damaged axons, triggering a signaling cascade from the axon to the cell body (5). This is followed by early cytoskeletal disruption (6, 7) and autophagy-mediated axonal degeneration (6), ultimately resulting in the progressive degeneration of axons distal to the injury site (8). The optic nerve, as a critical component of the central nervous system (CNS), faces significant barriers to regeneration following injury. In response to CNS injury, glial cells form scar tissue, and reactive astrocytes produce and release chondroitin sulfate proteoglycans, along with myelin-associated inhibitory factors (9), which impede axonal regeneration by disrupting the extension and navigation of growth cones (10). Moreover, the extracellular matrix (ECM) within the CNS influences cellular adhesion, migration, and signaling, presenting complex inhibitory properties that further hinder regeneration (11).

Prior research has demonstrated that the alteration of specific genes in RGCs markedly improves the axonal regeneration potential of mature RGCs. While these genes have been demonstrated to regulate optic nerve regeneration, the majority do not facilitate long-distance axon growth, preventing the axons from forming connections with brain structures including the suprachiasmatic nucleus (SCN), lateral geniculate nucleus (LGN), superior colliculus (SC), and other visual regions (12). Gradually, combinatorial techniques designed to prolong the regeneration of axons, including Zymosan/cAMP/PTEN deletion (13), CNTF/PTEN deletion/SOCS3 deletion (14), and UTX/PTEN (15), have produced encouraging regenerative results. During regeneration, RGC axons typically extend directly towards the brain but often demonstrate sudden U-turns, which we denote as “U turns.” Among the regenerating axons that reach the chiasm, the majority are located in the ipsilateral optic tract, indicating axonal misdirection, with just a minority crossing the midline into the contralateral optic tract or continuing into the undamaged optic nerve on the other side. There are considerable environmental disparities between the regions of the optic chiasm and the optic nerve, which present additional obstacles. The subsequent phase after long-distance optic nerve regeneration should concentrate on directing newly regenerated axons through the optic chiasm and into the brain (16).

## 2 Multifactorial combination inhibit axonal misguidance (U-turns) within the optic nerve

**Figure 1:**
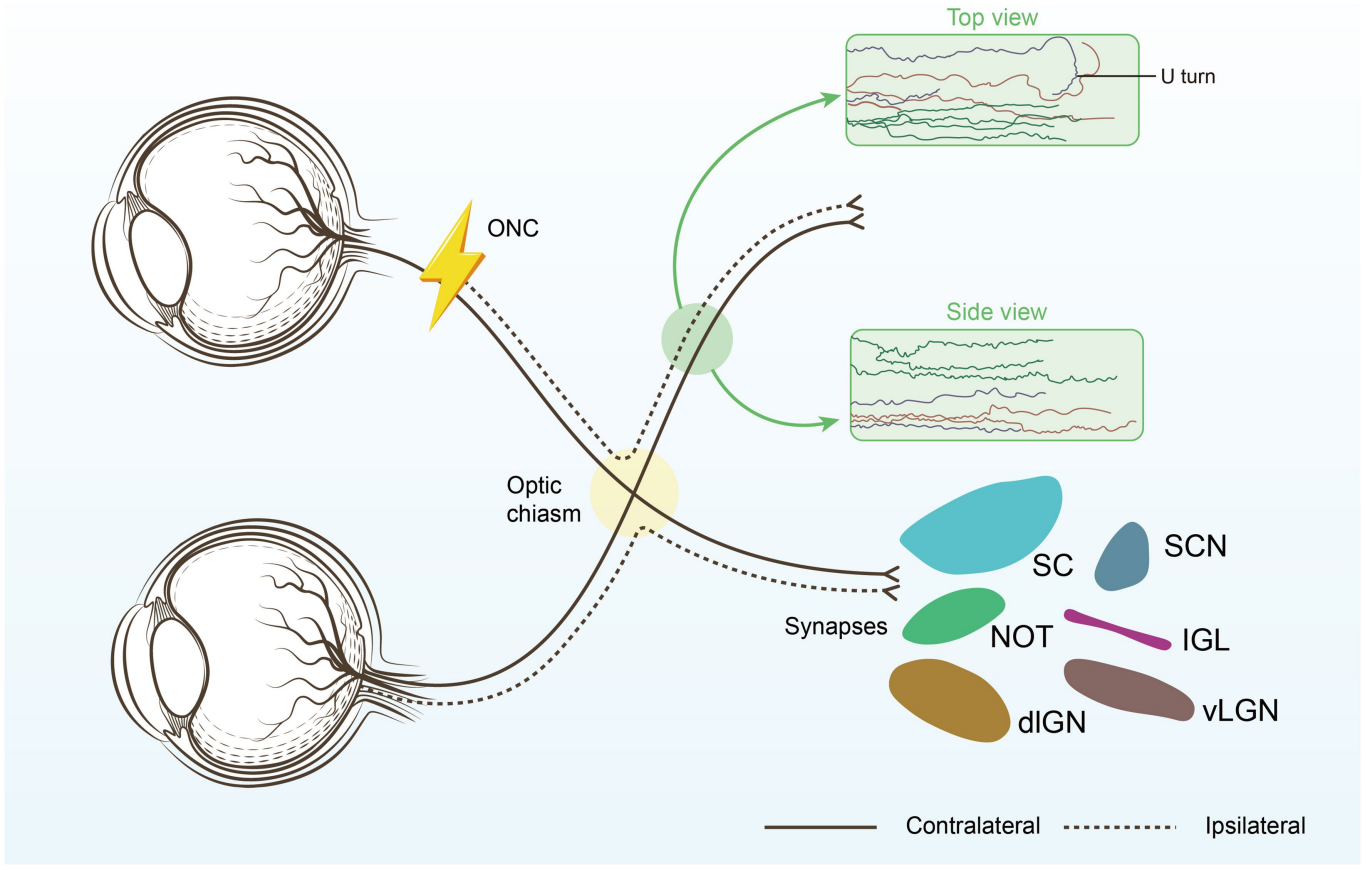
Restoring connections to the brain. The optimal repair strategy begins with promoting substantial long-distance regeneration of injured RGC axons, ensuring they reach their original targets. Next, the regenerating axons must be carefully guided through the optic chiasm to avoid U-turns and correctly reach their intended brain targets, each responsible for different aspects of visual processing. Suprachiasmatic nucleus (SCN), thalamic ventral or dorsal lateral geniculate nucleus (vLGN, dLGN), Intergeniculate leaflet (IGL), the nucleus of the optic tract (NOT), superior colliculus (SC).

Significant breakthroughs have been made in inducing axon regeneration in mature RGCs, successfully initiating the regeneration process. However, the next challenge is to correctly guide axon growth and establish new synapses and connections within the brain. Despite the initial success in inducing axon regeneration, many regenerated axons exhibit less-than-ideal growth patterns after reaching a certain length (12, 14, 17, 18), Optical nerves exhibit a bend in growth, known as a U-turn, when they are about to reach or have crossed the optic chiasm (Figure 1).

Tissue clearance and light sheet fluorescence microscopy (LSFM) are among the most precise methods for evaluating RGC axon regeneration, as they allow for clear assessment of axonal trajectories and growth patterns within the optic nerve (17). Our findings indicate that while RGC axons tend to project relatively straight towards the brain during regeneration, many axons take convoluted paths, with a significant number making abrupt U-turns. The proportion of U-turns is notably higher in proximal nerve regions compared to distal ones, with regions of high axonal misguidance likely corresponding to areas of intense astrocyte activation (17). The combination of tissue clearing techniques with retrograde trans-synaptic viral tracing provides a comprehensive and objective assessment of changes in retinal target areas receiving projections from RGCs following optic nerve injury. This approach may prove valuable for future evaluations of optic nerve injury and regeneration (19).

ROCK inhibition has a powerful effect on axon regeneration activated by AAV2.Stat3-ca. The suppression of axon regeneration is largely mediated by myelin-associated growth inhibitory proteins, which activate the Rho-A/ROCK pathway within neurons. To block ROCK in neurons, electric and magnetic stimulation can be effectively applied in the field of nerve regeneration. While stem cells are becoming a promising approach for regenerating RGCs, applied electric fields (EF) have been shown to guide RGC axons, with axons exhibiting cathode-directed growth in the presence of an EF. This effect is partially mediated by the Rho GTPase signaling cascade (20). Magnetic fields can induce currents, and the advantage of transcranial magnetic stimulation therapy is its ability to stimulate peripheral nerves and muscles without the pain caused by electrical stimulation (ES) and without the need for electrode placement (21, 22).

Following the administration of the cell-permeable ROCK inhibitor Y27632 into the vitreous chamber at the moment of injury and again 7 days later, subsequent analysis of axon regeneration 2 weeks post-injury revealed significant morphological alterations: the regenerated axons exhibited increased straightness, reduced deviation from the nerve pathway, and a decrease in U-turn frequency from 43% in the AAV2.Stat3-ca group decreased to 16% in the AAV2.Stat3-ca/Y27632 cohort. The concurrent application of AAV2.Stat3-ca and Y27632 similarly affected axon branching (23). In UTX/PTEN double-knockout mice, many regenerated axons reached and entered the optic chiasm, and the rate of U-turns was significantly lower compared to PTEN knockout alone (15). A chemotactic CXCL12/CXCR4-dependent mechanism traps growth-stimulated axons at the injury site, thereby limiting axon extension within the nerve. The CXCL12-mediated attraction causes axons to return to the injury site, but specific depletion of CXCR4 in RGCs reduces aberrant axon growth and enables long-distance regeneration (24).

Using nanoimprinting technology, a scaffold mimicking the microstructure of *in vitro* tissue was created to guide the growth and orientation of RGC axons. Axons derived from human-induced pluripotent stem cell (iPSC) RGCs elongated along the grooves of the scaffold, demonstrating effective guidance (25). Promoting long-distance regeneration of axons in damaged RGCs to ensure their arrival at the original target is a critical initial step in reparative strategies. Subsequently, the regenerated axons must be precisely guided through the optic chiasm (OX), avoiding U-turns, and correctly reaching the predetermined target areas in the brain responsible for various aspects of visual processing, which will be the focus of our subsequent research. Advances in ocular imaging technology have greatly expanded our understanding of mitochondrial retinopathies and optic neuropathies.

## 3 Combination of multiple factors can promote optic nerve regeneration

### 3.1 Mitochondrial modulation

Mitochondrial dysfunction has been implicated in the damage of retinal RGCs in optic neuropathies, as evidenced in Leber’s Hereditary Optic Neuropathy (LHON) and Autosomal Dominant Optic Atrophy (ADOA). The regeneration of the optic nerve necessitates an energy supply, and there is a clear correlation between the intracellular distribution of mitochondria and the process of nerve regeneration. In Optic Nerve Crush (ONC), there is a reduction of mitochondria in the dendrites during the retraction of dendrites, followed by an enlargement of mitochondria in the optic nerve/optic tract during axonal regeneration. A temporary increase in mitochondrial fission and biogenesis occurs in the RGCs’ cell bodies as the retinal dendrites regrow, with mitochondria transferring from dendrites to axons and then back again (26). Exogenous mitochondrial transplantation may be a promising approach to slowing the progression of neurological diseases. In a mouse model of ocular hypertension, an increase in the mitochondrial-free area within RGC axons and a reduction in mitochondrial transport with age have been observed. By transplanting mitochondria isolated from the liver into the vitreous body, the results showed a promotional effect on the survival and axonal growth of RGCs after ONC (27).

Mitochondria-targeted treatments have shown encouraging outcomes in facilitating optic nerve regeneration. M1, a tiny chemical that facilitates mitochondrial fusion and transport, can increase the length of mitochondria in peripheral axons of the sciatic nerve, as well as the motility and transport speed of axonal mitochondria *in vitro*. Subsequent to ONC, M1 markedly augmented the quantity of regenerated axons, which extended via the superior colliculus into several subcortical areas. This intervention reinstated local field potentials in the superior colliculus following optogenetic activation of RGCs, completely restored the pupillary light reflex, and facilitated the recovery of responses to low-intensity visual stimuli (28). When the key mitochondrial genes Opa1 or Mfn2 are knocked down, the growth-promoting effect of M1 is significantly neutralized, confirming the role of mitochondria in optic nerve regeneration. Multiple OPA1 isoforms form intricate homo-oligomers that constitute the structure of the mitochondrial cristae, thereby forming the entire mitochondrial network, which is closely related to optic nerve atrophy (29).

Mitochondrial dynamics are regulated by gene expression, and the mitochondrial protein armadillo repeat containing X-linked 1 (ARMCX1) plays an important role in the anchoring process during mitochondrial transfer. When ARMCX1 is overexpressed, it can effectively promote optic nerve regeneration (30). A pro-mitochondrial fission protein in the central nervous system, MTP18, the knockdown of which promotes axonal growth (31), serves as a downstream molecule of neuregulin, regulated by KLF7 and KLF9. The combined application of mitochondrial gene regulation and neurotrophic factors such as CNTF, which promotes nerve growth, also yields good regenerative results, once again proving the important role of regulating mitochondrial dynamics in optic nerve regeneration.

### 3.2 Activating the immune system to promote optic nerve regeneration

#### 3.2.1 Glial cell modulation

Following ONC, there is a substantial depletion of oligodendrocytes in adult murine models. To restore normal neural conduction following central nervous system injury, oligodendrocyte precursor cells must swiftly multiply, develop into mature oligodendrocytes, and myelinate regenerating axons. Astrocytes differentiate into two separate subsets: C3-positive and C3-negative reactive groups. Neuroprotective astrocytes generally function upstream in this neurotoxic pathway, obstructing the recruitment of detrimental microglia and macrophages, while mitigating the mortality of C3-positive neurotoxic astrocytes and neurons (32). p21 is a direct major regulator of reactive astrocyte proliferation. Neuroprotective astrocytes can inhibit the activation of microglia and the differentiation of downstream neurotoxic astrocytes, thereby promoting neural regeneration (32). In injured optic nerves, oligodendrocyte Precursor Cells (OPCs) undergo a brief proliferation but fail to differentiate into mature oligodendrocytes capable of myelination. Inherent GPR17 signaling in OPCs and sustained activation of microglia inhibit different stages of OPC differentiation. Manipulation of GPR17 and microglia can lead to widespread myelination of regenerating axons (33).

The astrocytic yes-associated protein (YAP) is pivotal in neuroinflammation. The conditional deletion of YAP in astrocytes results in exacerbated inflammatory infiltration and demyelination in the optic nerves of experimental autoimmune encephalomyelitis (EAE) mice, along with damage to RGCs. Astrocyte YAP can inhibit neuroinflammatory infiltration and demyelination by enhancing TGF-*β* signalling (34). BDNF, a factor secreted by astrocytes, has neuroprotective effects on both RGC axons and somata but does not affect axonal regeneration. Intravitreal injection of adenoviral vectors containing the BDNF gene (Ad.BDNF) into adult rats results in selective expression of the transgene by Müller cells, enhancing survival (35). New targeted gene therapy approaches have garnered significant attention. By using AAV vectors targeted at glial cells, damaged retinal neurons can be protected. CNTF released from glial cells has been shown to support the regeneration of optic fibers after optic nerve crush, allowing them to extend to the optic chiasm, with regenerated axons surviving in the injured optic nerve for at least 6 months (18). Additionally, the use of neuroprotective astrocyte-targeted AAV5 vectors directed at the optic nerve head has been explored to balance neurotoxic and neuroprotective astrocytes by modulating soluble adenylyl cyclase. This strategy has demonstrated that neuroprotective astrocytes can inhibit microglial activation and prevent the differentiation of downstream neurotoxic astrocytes, thereby promoting the survival of RGCs (32).

Following ONC, mobile zinc (Zn^2+^) accumulates in amacrine cells, which is thought to exacerbate microglial activation. Intravitreal injection of Zn^2+^ chelators, which inactivate Zn^2+^, has been shown to promote axon regeneration and increase RGC survival (36). Additionally, conditionally knocking out the zinc transporter 3 (ZnT3) in amacrine cells or RGCs further enhanced RGC survival and axon regeneration, leading to improved functional outcomes (36).

#### 3.2.2 Neutrophils promotes the survival and axonal regeneration of RGCs

Neutrophils are the primary responder of the innate immune system and are activated by injury. Pro-inflammatory leukocytes infiltrating the central nervous system are detrimental in multiple sclerosis and neuromyelitis optica, as well as Alzheimer’s disease and stroke (37, 38). Recent research indicates that granulocytes with characteristics of immature neutrophils accumulate in the vitreous fluid and exert neuroprotective and axonal growth effects by secreting a cocktail of growth factors (39). Manipulating the immune environment within the vitreous body can significantly impact the survival of RGCs and axonal regeneration after optic nerve crush (ONC). Oncomodulin (Ocm) is a calcium-binding protein secreted by activated macrophages and neutrophils. Immune clearance of neutrophils reduces Ocm levels in the retina, which inhibits axonal regeneration. A sharp decline in regenerative capacity follows the depletion of neutrophils. However, neutrophils typically stimulate the release of relevant growth factors from other cells, affecting subsequent inflammatory cascades (40). The co-injection of fluvastatin and MBV into the vitreous body induced strong protection and axonal regeneration of RGCs. Flow cytometry analysis revealed that the infiltration of neutrophils was significantly stronger than that in the control group. After blocking the action of neutrophils, the regenerative effect of the optic nerve was significantly reduced, indicating that neutrophils played an important role. However, this effect was transient and usually did not last more than 7 days (41).

In addition, the efficacy of CNTF gene therapy requires the activation of neutrophils. Ly6G is a neutrophil-specific surface protein, and the depletion of neutrophils through systemic injection of Ly6G antibodies significantly weakens the regenerative effects of CNTF, with a reduction in axonal regeneration (42). Selectively targeting neutrophils with anti-Ly6G can adjust the proportion of neutrophil subtypes, preserving the blood-retinal barrier and promoting RGC regeneration (43), and the functional knockout of complement receptor 3 can reduce ocular inflammation and protect the blood-retinal barrier. The combination of Ly6G+ bone marrow cells with recombinant IL-4 and G-CSF shows consistent cell surface phenotypes and transcriptomic features with immature neutrophils, which may become a new method for neuroregeneration treatment.

### 3.3 Coordinating lipid metabolism to promote axonal regeneration

Optic nerve regeneration is a complex process involving the precise regulation of multiple intracellular signaling and metabolic pathways. Lipid metabolism plays an essential role in optic nerve regeneration, as lipids are the main components of cell membranes and axonal myelin sheaths, and they are also an important source of cellular signal transduction and energy supply. Promoting axonal regeneration by regulating lipid metabolism is a very promising strategy. Neurons require a large amount of lipids to form cell membranes during regeneration (44, 45). There are many lipids with different functions in neurons, and not all lipids play a role in axonal growth. Depletion of neuronal lipin1 promotes axonal regeneration by regulating glycerolipid metabolism ^62^. Studies on neurons of Drosophila larvae have revealed the function of phospholipid balance in dendritic morphogenesis (46). Triglycerides may provide lipid precursors to generate Phospholipids (PLs) for membrane phospholipids during axonal regeneration. Currently, there is relatively little understanding of how lipid metabolism in neurons controls axonal elongation and regeneration.

After optic nerve injury, the reprogramming of lipid synthesis within neurons significantly affects regenerative capacity. Specifically, the injury leads to the upregulation of lipin1, a key enzyme in lipid synthesis, which drives neurons to preferentially synthesize triglycerides. By inhibiting the expression of lipin1, steering lipid synthesis in neurons from triglycerides to phospholipids can significantly promote axonal regeneration (45). Blocking the interaction between Neogenin and its ligand RGMa can reduce the localization of Neogenin in lipid rafts, thereby lifting the inhibitory effect on axonal growth. This strategy has not only demonstrated effective repair of optic nerve injuries in the laboratory but also shows potential for restoring motor function in spinal cord injury models (47).

### 3.4 Neurotrophic factor combination therapy

Activation of NF signalling represents a possible therapeutic strategy for combating neurodegeneration. Neurotrophic factors constitute a group of diffusible proteins that are essential for the growth, differentiation, and development of neuronal cells (48). In the field of optic nerve regeneration, neurotrophic factors such as ciliary neurotrophic factor (CNTF), brain-derived neurotrophic factor (BDNF), and glial cell line-derived neurotrophic factor (GDNF) have been extensively studied (49) and are leading therapeutic candidates for promoting neuroprotection and axon regeneration after CNS injury (50). However, recombinant CNTF has shown limited effectiveness in promoting axon regeneration (51). BDNF is one of the most effective factors for enhancing RGC survival after axotomy (52), though it cannot completely prevent RGC death. The effects of CNTF are closely linked to cAMP levels, inflammatory stimuli, and STAT3 pathway activation. The limited efficacy of CNTF appears to be due to the increased expression of SOCS3, which inhibits the Jak–STAT signaling pathway as RGCs mature. SOCS3 deletion restores the responsiveness of RGCs to CNTF (51, 53, 54). In recent years, CNTF has often been combined with gene therapy to promote repair after injury (49).

Although the optic nerve typically does not regenerate after injury or in degenerative diseases such as glaucoma, this failure can be partially reversed by inducing a controlled immune response within the eye. Inflammatory stimuli have been shown to activate RGCs into an active regenerative state (55). Stromal cell-derived factor 1 (SDF1) enhances the axon growth-promoting effects of the myeloid-derived protein oncomodulin (Ocm), which is expressed by infiltrating myeloid cells (56). The combination of cAMP with SDF1 can further amplify Ocm’s neuroregenerative effects (13). Oncomodulin is a calcium-binding protein secreted by activated macrophages and neutrophils in the vitreous and retina. Lens injury, injection of dextran, or other inflammatory conditions trigger a massive influx of inflammatory cells, producing high levels of oncomodulin, which plays a crucial role in axon regeneration following lens injury (57). Lens injury has neuroprotective effects on RGCs, promotes axon growth, and partially alleviates inhibitory conditions. Preconditioning with lens injury 2 weeks before optic nerve crush (ONC) increases optic nerve regeneration threefold and enhances RGC survival (58). However, in CNTF or LIF knockout mice, the regenerative effects of lens injury are significantly reduced (55).

### 3.5 Extracellular matrix combined therapy

In a study on the ECM of spinal cord cells, the decellularized spinal cord ECM was extracted from newborn (DNSCM) and adult (DASCM) rabbits and subjected to differential analysis. It was found that decellularized newborn spinal cord matrix (DNSCM) not only exhibits superior performance as neural progenitor cells (NPCs) and organoids in spinal cord injury (SCI) models but also more effectively promotes the proliferation, migration, and neural differentiation of NPCs, as well as the axonal growth of spinal cord organoids compared to decellularized adult spinal cord matrix (DASCM) (59). Similar to optic nerve development, there must be certain differences in the extracellular components of the optic nerve at the neonatal stage. For instance, pleiotrophin (PTN) and tenascin (TNC) in DNSCM are considered to play important roles in the spinal cord ECM, which are lacking in the mature ECM. Unlike the spinal cord, the cell bodies of RGCs may be regulated by the ECM within the retina, while the axons surrounded by the axons may be regulated by the ECM within the optic nerve sheath. There is currently no clear research on the ECM at different stages.

**Figure 2:**
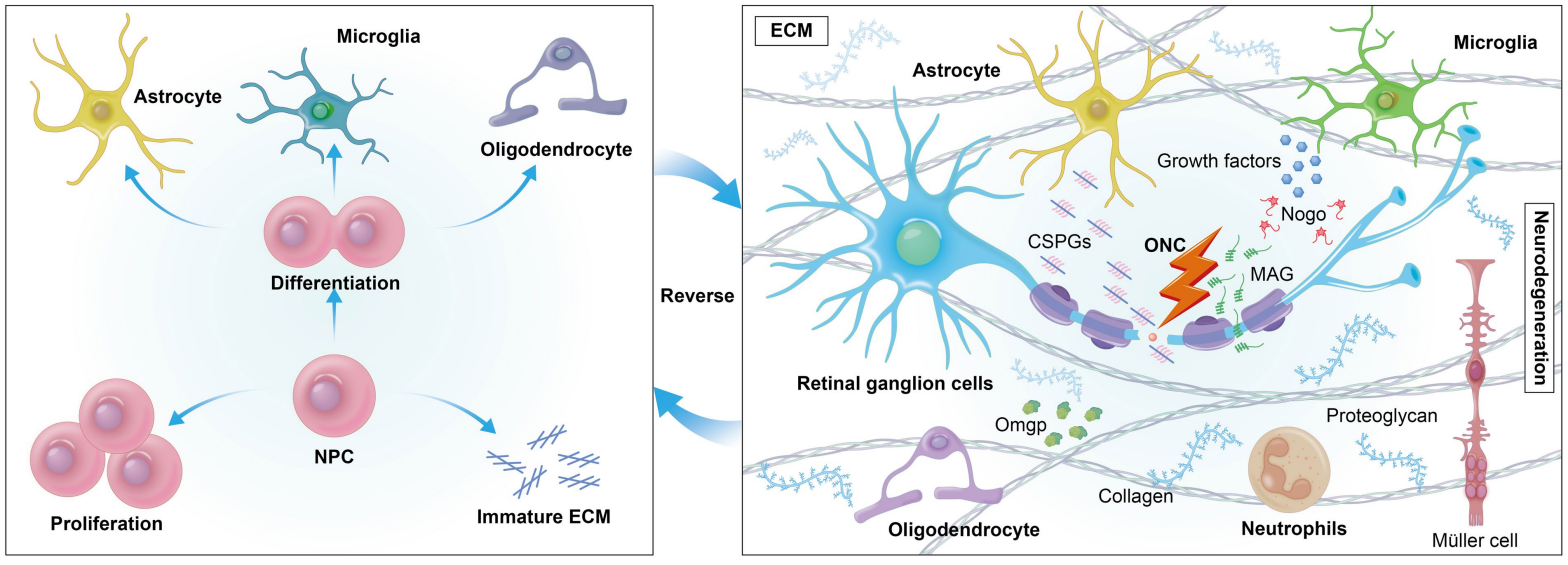
Extrinsically, cells encounter a variety of growth-inhibitory factors in their environment, such as myelin-associated inhibitors (MAIs) and inhibitory proteoglycans. The formation of an inhibitory environment rich in CSPGs after CNS injury is a primary obstacle. Reversing mature ECM may foster a regenerative environment by reducing inhibitory factors like CSPGs, promoting axonal growth.

Extracellular growth-inhibitory factors, such as chondroitin sulfate proteoglycans (CSPGs), present significant challenges to axonal regeneration. After central nervous system (CNS) injury, CSPGs, which are part of the scar tissue, form an inhibitory environment that hinders optic nerve regeneration by binding to neuronal receptors and promoting an astrocyte-secreted inhibitory matrix that impedes nerve regeneration (60–63). Myelin-associated substances like Nogo, myelin-associated glycoprotein (MAG), and oligodendrocyte-myelin glycoprotein (OMgp) also interact with neuronal receptors to activate the RhoA/ROCK signaling cascade, further impeding axonal development (64–66). The ECM is essential in tissues like the retina and optic nerve, providing a dynamic network for remyelination support and cellular regulation. The accumulation of CSPGs post-injury often leads to an inhibitory environment that can be mitigated by deleting protein tyrosine phosphatase sigma (PTPσ), which reduces neuronal sensitivity to CSPGs and allows regenerating RGCs axons to penetrate the glial scar at the injury site (67, 68). Furthermore, modulating the interaction between amacrine cells (ACs) and RGCs, especially through dopamine receptor activation or by increasing dopamine release with levodopa in dopaminergic amacrine cells (DACs), has shown neuroprotective effects and moderately improved axon regeneration (69). These findings underscore the complex interplay between various cellular components and the ECM in neural regeneration, highlighting potential therapeutic targets for overcoming the inhibitory effects of the CNS injury environment. Neighboring cells such as astrocytes, Müller glia, microglia, and neutrophils play a crucial role in neural regeneration (Figure 2).

In an ischemic retinal model, the expression of ECM-related proteins such as fibronectin, laminin, and tenascin C in the retina and optic nerve of ischemia/reperfusion rat models was analyzed. The ECM components are dysregulated in the retina and optic nerve, with fibronectin showing significantly elevated mRNA and protein levels in the ischemic retina, while laminin and tenascin C exhibit enhanced immunoreactivity in the ischemic optic nerve (70).

### 3.6 Electromagnetic stimulation induces regeneration direction

Effective directional axonal development and neural cell migration are essential for neural regeneration in the central nervous system (CNS) when the length of optic nerve regeneration extends to the optic chiasm or beyond. Appropriate guidance is essential for the restoration of the entire optic nerve projection system. Applied direct current (DC) electric fields (EFs) can direct axonal growth *in vitro*, and *in vivo* studies have sought to improve the regeneration of injured spinal cord axons utilising DC electric fields (71–73). Exploring effective methods for neural cell migration and directional regeneration, as well as understanding their underlying mechanisms, is of great significance. Endogenous electric fields (EFs) are widely present in the developing nervous system and play an important role in CNS development. The presence of electric currents may influence cellular behavior during nervous system development, depending on the size, location, and timing of the electric fields (74–77). When endogenous electric fields are selectively disrupted, developmental defects in neural structures such as the neural tube, notochord, and somites can be observed (78). Electrical stimulation has also been shown to assist in the survival of RGCs, with the application of exogenous EFs immediately after optic nerve transection resulting in an RGC survival rate 1.5 times higher than that of the control group (79). A significant biological effect of electric fields is the induction of directional cell migration. Applied electric fields have profound effects on neurite growth as well as the migration of neurons and stem cells. Both endogenous and applied electric fields may serve as guiding signals, aiming to activate endogenous transcription and molecular signaling pathways (80) to guide neural tissue regeneration and enhance functional connectivity (81, 82). Electric fields can activate voltage-sensitive calcium channels, and the influx of calcium can initiate transcriptional programs that trigger important intracellular signaling pathways related to regeneration. Inhibitors of Rho GTPase signaling can neutralize this effect (83). However, these techniques have not yet reached clinical treatment, as DC current can cause charge accumulation and oxidative tissue damage, though electrical stimulation remains a feasible approach for directing optic nerve regeneration.

In recent years, magnetic stimulation, particularly repetitive transcranial magnetic stimulation (rTMS), has shown potential as a non-invasive brain stimulation technique in neural repair. rTMS generates a time-varying magnetic field through coils placed on the scalp, which induces electrical currents beneath the cortex via Faraday induction, thereby modulating neural activity. rTMS has been widely applied in the treatment of neurological and psychiatric disorders, with therapeutic effects lasting from hours to days (21). Tang et al. used low-intensity repetitive transcranial magnetic stimulation (LI-rTMS) to study its effects on the survival of RGCs and axonal regeneration in a mouse optic nerve crush injury model (84). The study found no significant improvement in RGC survival compared to the control group, nor was axonal regeneration observed. Although existing research indicates that low-intensity rTMS has limited direct effects on optic nerve regeneration, it still holds potential in modulating neural plasticity and promoting neural circuit reorganization in healthy tissue. Future research should explore the application of high-intensity rTMS to maximize its neuroregenerative effects. Additionally, the combined use of rTMS with other treatments, such as electrical stimulation, should be investigated to achieve better therapeutic outcomes at different stages of neural injury.

## 4 Epigenetic and protein modifications in long-distance regeneration

### 4.1 DNA methylation regulates robust regeneration

Regulating DNA methylation is crucial in epigenetic modifications; it alters the compact chromatin structure, thereby releasing the cell’s transcriptional capacity (85, 86). The TET enzyme family, responsible for DNA demethylation, regulates optic nerve regeneration with TET1 and TET2 being critical in this process (46, 87, 88). The ectopic expression of Oct4, Sox2, and Klf4 genes (OSK) in mouse RGCs can reinstate youthful DNA methylation patterns and transcriptome, thereby enhancing axonal regeneration post-injury and reversing visual impairment in a glaucoma mouse model and elderly mice. This epigenetic reprogramming results in an increase in axonal density compared to control mice not treated with OSK (89), highlighting the potential of epigenetic modifications in facilitating long-distance axonal regeneration. X-chromosome associated gene encoding a histone demethylase, as a new regulator of neural regeneration in mammals. UTX has been shown to inhibit spontaneous axon regeneration in the peripheral nervous system (PNS), and when knocked out or pharmacologically inhibited in retinal ganglion cells (RGCs) of the central nervous system (CNS), it significantly enhances neuronal survival and optic nerve regeneration (15). DNA methyltransferase 3a (DNMT3a) significantly inhibits axonal regeneration in mouse and human retinal explants. By genetically regulating the selective suppression of DNMT3a expression in RGCs, the reactivation of the regenerative genetic program is achieved, leading to a robust regeneration response.

### 4.2 Histone modifications

Histone deacetylases (HDACs) become more active following optic nerve injury, contributing to the death of RGCs (90). In particular, the activity of HDAC3 significantly increases after injury, leading to the deacetylation of histone H4 and the suppression of gene expression relevant to regeneration (91, 92). Oxidative stress, present in both the early and sustained stages after optic nerve injury, causes damage to RGCs and further inhibits axonal regeneration through multiple pathways. HDAC inhibitors have been shown to promote nerve regeneration by mitigating oxidative stress-induced damage to RGCs. Additionally, inflammatory responses can further suppress regeneration by upregulating HDAC3 (93, 94). Histone methyltransferases establish repressive chromatin structures by catalyzing the trimethylation of histone H3 at lysine 27 (H3K27me3). Overexpression of Ezh2 in RGCs within the central nervous system facilitates optic nerve regeneration via both histone methylation-dependent and methylation-independent pathways. Ezh2 promotes axonal regeneration by coordinating the transcriptional repression of genes that regulate synaptic function and impede axonal regeneration, while simultaneously activating several factors that enhance axonal regeneration (94).

### 4.3 mRNA modification

N6-methyladenosine (m6A) plays a crucial molecular role in RNA maturation and is the most abundant internal modification of mRNA, including RNA splicing, localization, decay, and translation (95). Changes in m6A modification detected after optic nerve crush (ONC) indicate that genes related to m6A, such as METTL3, WTAP, FTO, and ALKBH5, are all upregulated post-injury (96). An enzyme called Alkbh5 removes the m6A modification and controls the amount of axonal regeneration after peripheral nerve injury. In the brain and spinal cord, decreasing the expression of Alkbh5 can improve the survival rate and axonal regeneration capacity of RGCs in mice after an eye injury (97). Specific ribosomal interacting proteins, such as huntingtin (HTT), can selectively control the translation of specific subsets of mRNAs. Selective translation through the customization of translational complexes is a key mechanism for axonal regeneration and holds significant importance for the development of therapeutic strategies for central nervous system repair (98).

### 4.4 Ubiquitination in optic nerve regeneration

Ubiquitination is essential for numerous physiological processes, including cell survival, differentiation, and both innate and adaptive immunity. It is a dynamic, multidimensional post-translational alteration that encompasses nearly all elements of eukaryotic organisms and significantly influences human development, disease, and ageing (99). Ubiquitination begins with the attachment of a single ubiquitin molecule to the lysine residue of a target protein. Ubiquitin is conjugated to target proteins via three sequential steps: activation by ubiquitin-activating enzymes (E1s), conjugation by ubiquitin-conjugating enzymes (E2s), and ligation by ubiquitin ligases (E3s) (99). Once attached to the substrate, the 76-amino acid ubiquitin protein undergoes further modifications, generating a variety of distinct signals that produce different cellular outcomes (100). The ubiquitin proteasome system (UPS) effectively addresses various proteostasis requirements by coordinating the functions of over 500 components, removing misfolded or damaged proteins, facilitating receptor signalling pathways, responding to DNA damage and oxidative stress, and promoting the cell cycle (101). The UPS is required for the dynamic remodeling of synaptic structures following synaptic activity (102). Pharmacological inhibition of the UPS leads to a marked reduction in activity-dependent synaptic plasticity and a dose-dependent loss of synaptic connections (103). Mitochondrial activity is essential for brain regeneration and is intricately linked to the process of ubiquitination. Mitochondria possess their own genome; nevertheless, the majority of mitochondrial proteins are encoded by the nuclear genome, synthesised by cytosolic ribosomes, and subsequently transported from the cytosol into the mitochondria (104). Since the identification of the UPS, many key cellular metabolic pathways, including the AMPK pathway, have been found to be modulated by the UPS (105). Recent studies have shown that the depletion of cellular amino acids alone or in combination leads to the ubiquitination of mTOR, thereby inhibiting the activity of mTORC1 kinase through the GCN2-FBXO22-mTOR pathway by preventing substrate recruitment (106). During the process of cardiac fibrosis, PDCD5 is upregulated by SMAD3, and PDCD5 promotes the ubiquitination of HDAC3, thus inhibiting it, and subsequently improves progressive fibrosis and cardiac dysfunction by inhibiting HDAC3 (107). The inhibition of HDAC3 also helps promote neuronal regeneration, but this has not yet been verified in models of optic nerve injury (93, 94). Apoptosis of photoreceptors is an important pathogenic mechanism of retinal degeneration. Hypoxia-induced hypoxia-inducible factor-1α (HIF-1α) activation prevents the ubiquitination and degradation of GAP43 mediated by the tripartite motif protein 25 (TRIM25), leading to the upregulation of GAP43 in Hyp-sEVs, thus achieving the effect of photoreceptor protection (108).

## 5 Multi-gene combined therapy promotes long-distance optic nerve regeneration

**Figure 3:**
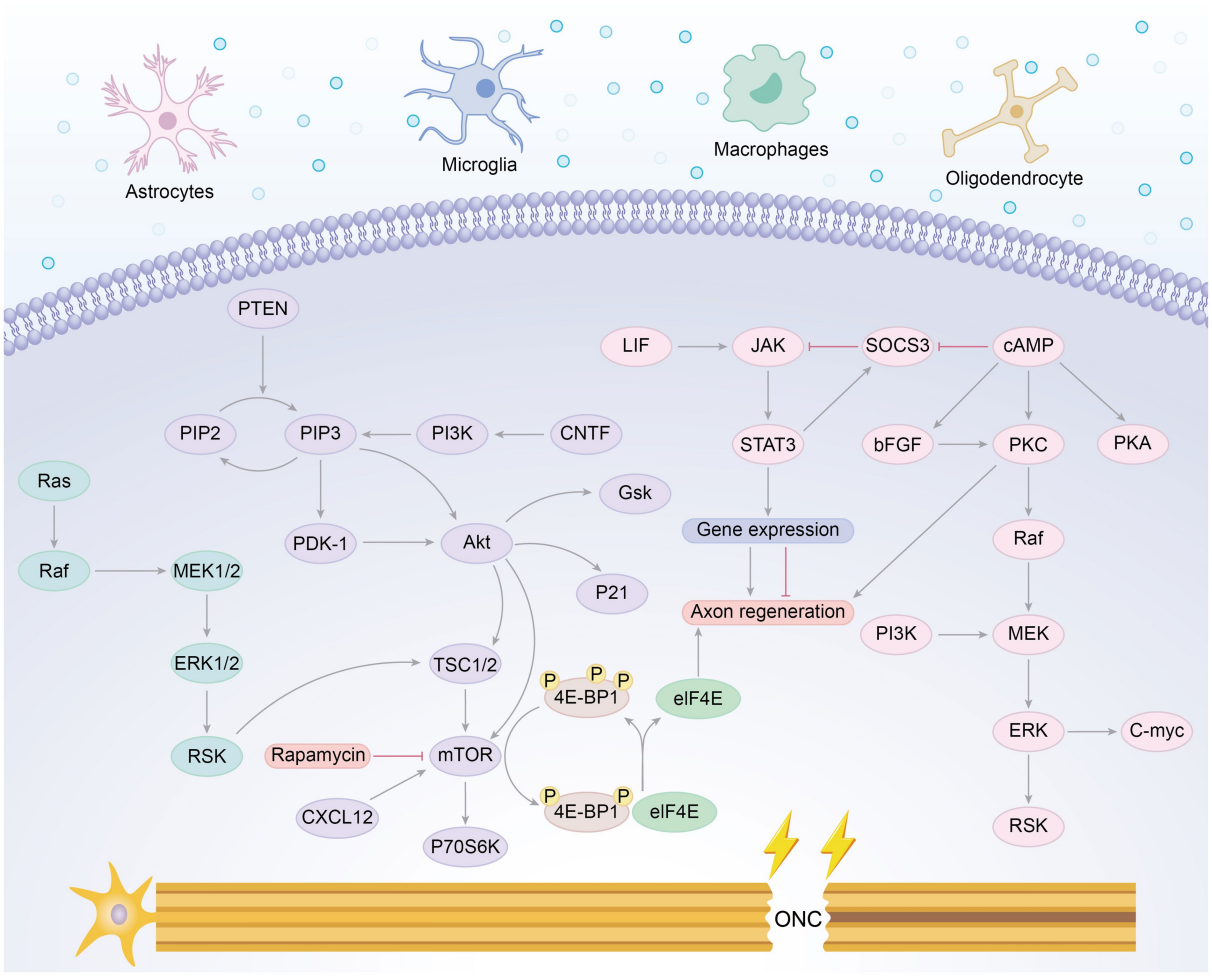
Signaling pathways involved in axon regeneration after optic nerve injury. Successful axonal regeneration involves intrinsic pathways of RGCs and factors from surrounding cell types. The schematic illustration highlights key regulatory factors and their associated downstream signaling pathways that are involved in the intrinsic growth mechanisms driving axon regeneration following optic nerve injury.

In the early stages of visual system development, RGCs rapidly extend their axons both *in vivo* and in culture, and they can achieve short-distance regeneration after injury (109, 110). However, this capacity for axonal growth and regeneration declines postnatally, with the damaged optic nerve struggling to regenerate due to various intrinsic neuronal limitations and external environmental barriers, leading to irreversible vision loss (45, 109, 111, 112). RGCs exhibit markedly different regenerative capacities at various developmental stages, with those during the embryonic period or just after birth exhibiting stronger regenerative capabilities (109). The ECM during early development may significantly enhance the regenerative capacity of the central nervous system (59), and the activation of specific signaling pathways, such as the MAPK cascade, is critical for injury response (33). Genetic manipulation via multiple signaling pathways, including the PTEN/mTOR pathway, JAK/STAT3 pathway, KLF pathway, Sox11 pathway, and RhoA/ROCK pathway, has shown notable advancements in optic nerve axon regeneration (12, 113). Combination therapies utilizing diverse routes have shown synergistic effects, enhancing regenerative outcomes in optic nerve regeneration (12, 113). Despite this, only a small subset of PTEN-deleted RGCs actually regenerate, while most surviving RGCs still lack regenerative capacity (114). PTEN deletion remains the most effective single-gene manipulation strategy, and combining it with other signaling molecules is crucial for developing clinically viable neural repair strategies (12, 114, 115). For instance, combining zymosan, CPT-cAMP, and PTEN deletion has resulted in robust long-distance axon regeneration, allowing axons to extend from the back of the eye through the entire optic nerve and cross the optic chiasm (13). Additionally, combining Anxa2 and tissue plasminogen activator (tPA) with PTEN knockdown enables axons to cross the optic chiasm and grow into the optic tract 8 weeks post-optic nerve injury, with some axons even crossing into the contralateral optic nerve (114). Furthermore, simultaneous deletion of PTEN and SOCS3 in RGCs allows optic nerve axons to reinnervate the hypothalamus, forming new synapses with neurons in the suprachiasmatic nucleus (SCN) (116). The transcriptional function of STAT3 is crucial for CNS axon regeneration, and MEK regulates the localization and function of STAT3, with PTEN deletion enhancing its role in promoting optic nerve regeneration (117). Moreover, combining PTEN deletion with gene overexpression, such as B-RAF, DCLK2, and Sox11, has yielded promising regenerative outcomes (118–120). Identifying genes or pathways that protect RGCs or specific subtypes from cell death is equally important, as Sox11 promotes regeneration in non-*α*-RGCs, which are resistant to PTEN deletion-induced regeneration (2, 120). Figure 3 illustrates the signaling pathways involved in axon regeneration after optic nerve injury, highlighting the key regulatory factors and their associated downstream signaling pathways that are integral to the intrinsic growth mechanisms driving axon regeneration.

One of the genes induced after JAK/STAT3 activation is SOCS3 (suppressor of cytokine signaling 3), which inhibits the JAK/STAT3 pathway by acting on gp130 (121, 122). Elimination of SOCS3 results in pathway activation and significant axon regrowth. Researchers have found that the concurrent elimination of PTEN and SOCS3 produces a synergistic effect, markedly improving both the strength and durability of axon regeneration (116). PTEN acts as a negative regulator of the mTOR pathway, while SOCS3 negatively regulates the JAK/STAT3 pathway. Furthermore, substantial axon regeneration beyond the superior colliculus has been observed in animals treated with PTEN and SOCS3 gene deletions combined with c-Myc gene overexpression (14). Co-overexpression of c-Myc and CNTF, alongside PTEN and SOCS3 co-deletion, allows regenerated optic nerve axons to cross the optic chiasm and extend within the optic tract (123). Additionally, drugs that elevate cAMP levels have been shown to enhance CNTF- and immune-induced axon regeneration, possibly by suppressing SOCS3 expression through increased cAMP levels (53). We have summarized the current classic multi-gene combinations for long-distance optic nerve regeneration, as well as whether the length of regeneration reaches the optic chiasm and brain Regions (Table 1).

**Table 1:** Multi-gene combined therapy for promoting the survival and axonal regeneration of RGCs are listed.

Author	Year of publication	Gene	Interventions	Phenotype	Optic chiasm	Brain Regions restoration
Wang et al. (8)	2023	Pten/Socs3/ptpn2 + CNTF+IFNγ	Deletion	Promoted axon regeneration	Y	N
O’Donovan et al. (118)	2014	B-RAF/Pten	Expression/deletion	Promoted axon regeneration	N	N
Nawabi et al. (119)	2015	DCLK2/Pten	Expression/deletion	Promoted axon regeneration	Y	N
Li et al. (116)	2015	Pten and Socs3	Deletion	Promoted axon regeneration	Y	Y
Lim et al. (125)	2016	RHEB1/visual stimulation	Overexpression	Promoted axon regeneration	Y	Y
Li et al. (126)	2017	Zinc/Pten	Deletion	Promoted axon regeneration	Y	N
Norsworthy et al. (120)	2017	SOX11/Pten	Overexpression/deletion	Promoted axon regeneration	Y	Y
Yungher et al. (127)	2017	Bax/CNTF	Knockout	Promoted axon regeneration	Y	Y
Bennett Au et al. (28)	2022	Pten/M1	Deletion	Promoted axon regeneration	Y	N
Trakhtenberg (128)	2018	Zinc/Klf9	Knockdown	Promoted axon regeneration	Y	N
Kurimoto et al. (13), de Lima et al. (129), Luo et al. (17), and Goulart et al. (130)	2010 2012 2013 2018	Zymosan/cAMP/Pten	Deletion	Promoted axon regeneration	Y	Y
Sun et al. (14)	2011	Pten and Socs3/CNTF	Deletion	Promoted axon regeneration	Y	Y
Yungher et al. (131)	2015	Pten/cAMP/ CNTF	Knockdown/overexpression	Promoted axon regeneration	Y	Y
Luo et al. (117)	2016	STAT3 and MEK/Pten	Co-activation/deletion	Promoted axon regeneration	Y	Y
Leibinger et al. (132)	2017	CRMP2 and GSK3/Lens injury	Co-activation	Promoted axon regeneration	Y	N
Belin et al. (123)	2015	c-Myc/CNTF/Pten and Socs3	Overexpression/deletion	Promoted axon regeneration	Y	N
Li et al. (114)	2022	Anxa2/tPA/Pten	Knockdown	Promoted axon regeneration	Y	N
Jacobi et al. (122)	2022	Pten/Atf4	Knockdown	Promoted axon regeneration	Y	N
Xie et al. (133)	2023	Zymosan/ArmC10	Combination	Promoted axon regeneration	N	N
Xie et al. (56)	2022	Pten/SDF1/Zymosan/cAMP	Knockdown	Promoted axon regeneration	Y	N
Leaver et al. (134)	2023	BCL2/CNTF	Overexpression	Promoted axon regeneration	Y	N
Logan et al. (135)	2023	FGF/NT3/ BDNF	Combination	Promoted axon regeneration	N	N
Cen et al. (63)	2017	CNTF/RhoA silencing	Knockdown	Promoted axon regeneration	N	N
Wang et al. (94)	2023	Ezh2/omg2	Knockdown	Promoted axon regeneration	N	N
Wang et al. (136)	2018	Lin28a/Pten	Overexpression/Knockdown	Promoted axon regeneration	N	N

## 6 Discussion

Significant progress has been made in promoting the survival of RGCs and the regeneration of the optic nerve. However, new challenges have also emerged. Despite identifying many genes that can be manipulated to enhance optic nerve regeneration, our understanding of the cellular and molecular mechanisms regulating axon regeneration remains incomplete. The use of multifactorial strategies that combine different genes and cellular pathways offers new avenues for research.

The formation of new synapses and connections within brain tissue will be a key focus of future research. During early development, RGC axons grow rapidly, enabling them to extend from the retina to distant targets in the brain (124). However, as RGCs transition from an embryonic to a postnatal state, critical steps and signaling pathways that once supported axon growth begin to shut down. Unlike neurons in the peripheral nervous system (PNS), which can reawaken their intrinsic regenerative capacity, the central nervous system (CNS) fails to regenerate. Understanding the differences between these two systems will be crucial. The optic chiasm, a critical relay station between the retina and the brain, presents a significant challenge for nerve regeneration. When regenerating axons reach the optic chiasm, many make misdirected turns, preventing further extension. Investigating whether the optic chiasm presents an inhibitory environment and exploring ways to modulate this environment could help axons successfully cross the chiasm and reach the brain. Does the ECM within the optic chiasm share the same regenerative mechanisms as other regions? The immune microenvironment of the optic chiasm during regeneration, and whether targeted modulation of glial cells could improve this environment, remains an unexplored area.

Optic nerve injury is a multi-stage complex process involving various cell types and molecular mechanisms. At different stages of injury, changes in gene expression patterns and the structural components of the microenvironment occur, which are crucial for the repair and regeneration of the injury. Therefore, understanding the specific characteristics of each stage and developing targeted therapeutic strategies based on this is a current focus of research. In the early stages of optic nerve injury, which are usually caused by physical impact, ischemia, or other forms of trauma, cells may experience immediate mechanical damage, leading to ruptured cell membranes and destruction of the cytoskeleton. In addition, inflammatory responses are quickly initiated, and microglia and macrophages begin to accumulate in the area of injury, releasing inflammatory mediators such as cytokines and chemokines to clear damaged cells and debris. The therapeutic strategy at this stage may focus on controlling inflammatory responses and protecting cells from further damage. As the injury progresses to the middle stage, cell death and tissue destruction may intensify, while the potential for neural regeneration begins to emerge. At this stage, glial cells such as astrocytes and oligodendrocytes may be activated, forming a glial scar that limits the spread of injury to some extent but also hinders axonal regeneration. The treatment strategy may need to consider how to modulate the response of glial cells to reduce scar formation while promoting neural regeneration. In the late stage, the area of injury may stabilize, but the possibility of neural function recovery decreases. At this stage, the focus of treatment may be to promote neural regeneration and remodeling, as well as to improve neural function. This may involve stimulating axonal growth, promoting remyelination, and enhancing synaptic plasticity. In addition, it may also be necessary to consider how to support the survival and function of neurons through neurotrophic factors or other molecular means. In order to achieve the maximum salvage at different stages of injury, researchers are exploring various methods, including drug therapy, gene therapy, cell transplantation, and the application of biomaterials. These methods aim to achieve therapeutic effects by regulating inflammatory responses, promoting cell survival, enhancing neural regeneration, and improving neural function. However, due to the complexity of optic nerve injury, interdisciplinary collaboration and innovative research methods are needed to better understand the mechanisms of injury and develop effective treatment strategies.

Currently, the most commonly used animal model for studying optic nerve regeneration following injury is the optic nerve crush model. However, it is crucial to validate the efficacy of multifactorial gene therapy strategies in models of clinically relevant optic nerve diseases, such as glaucoma, optic neuritis, optic neuropathy, and optic atrophy. Although multifactorial gene therapy has shown promising regenerative effects in animal models, many challenges remain, including the efficiency of gene delivery, precise control of gene expression, and the safety of these therapies. Advances in gene editing technologies (such as CRISPR-Cas9) and gene delivery systems (such as AAV viral vectors) hold the potential for significant breakthroughs in clinical applications. Future research should continue to explore the optimal combinations of different genes and their applicability to various types of optic nerve injuries. Additionally, combining gene therapy with other treatment modalities, such as cell transplantation and pharmacotherapy, deserves further investigation to develop more effective and safer strategies for optic nerve regeneration.
